# Posttranscriptional regulation of adrenal TH gene expression contributes to the maladaptive responses triggered by insulin-induced recurrent hypoglycemia

**DOI:** 10.14814/phy2.12307

**Published:** 2015-02-23

**Authors:** Necla Kudrick, Owen Chan, Edmund F La Gamma, Juhye Lena Kim, Arnold William Tank, Carol Sterling, Bistra B Nankova

**Affiliations:** 1 The Regional Neonatal Center, Maria Fareri Children's Hospital at Westchester Medical CenterValhalla, New York; 2 Department of Internal Medicine, Section of Endocrinology and Metabolism, Yale School of MedicineNew Haven, Connecticut; 3 Division of Newborn Medicine, Departments of Pediatrics, Biochemistry and Molecular Biology, New York Medical CollegeValhalla, New York; 4 Department of Pharmacology and Physiology, University of Rochester Medical CenterRochester, New York

**Keywords:** Adrenal medulla, epinephrine, hypoglycemia-associated autonomic failure, tyrosine hydroxylase gene expression

## Abstract

Acute metabolic stress such as insulin-induced hypoglycemia triggers a counterregulatory response during which the release of catecholamines (epinephrine), the activation of tyrosine hydroxylase (TH) enzyme and subsequent compensatory catecholamine biosynthesis occur in the adrenal medulla. However, recurrent exposure to hypoglycemia (RH), a consequence of tight glycemic control in individuals with type 1 and type 2 diabetes compromises this physiological response. The molecular mechanisms underlying the maladaptive response to repeated glucose deprivation are incompletely understood. We hypothesize that impaired epinephrine release following RH reflects altered regulation of adrenal catecholamine biosynthesis. To test this hypothesis, we compared the effect of single daily (RH) and twice-daily episodes of insulin-induced hypoglycemia (2RH) on adrenal epinephrine release and production in normal rats. Control animals received saline injections under similar conditions (RS and 2RS, respectively). Following 3 days of treatment, we assessed the counterregulatory hormonal responses during a hypoglycemic clamp. Changes in adrenal TH gene expression were also analyzed. The counterregulatory responses, relative TH transcription and TH mRNA levels and Ser40-TH phosphorylation (marker for enzyme activation) were induced to a similar extent in RS, 2RS, and RH groups. In contrast, epinephrine and glucagon responses were attenuated in the 2RH group and this was associated with a limited elevation of adrenal TH mRNA, rapid inactivation of TH enzyme and no significant changes in TH protein. Our results suggest that novel posttranscriptional mechanisms controlling TH mRNA and activated TH enzyme turnover contribute to the impaired epinephrine responses and may provide new therapeutic targets to prevent HAAF.

## Introduction

Recent exposure to hypoglycemia impairs the behavioral, neuroendocrine, and autonomic responses to subsequent glucoprivic episodes, a condition which has been termed hypoglycemia-associated autonomic failure (HAAF) (Cryer 2013a). HAAF is a common complication in diabetic patients undergoing intensive insulin therapy and a major public health concern (Cryer 1992, 2006; Veneman et al. 1993; Diabetes Control and Complications Trial Research Group 1997). While the inability of diabetic patients to reduce exogenous insulin and increase glucagon secretion in response to hypoglycemia put them at greater risk for adverse metabolic outcomes, the clinical syndrome of HAAF develops only when the sympathoadrenal and symptomatic responses become attenuated (Cryer 2013a). Several hypotheses attribute the blunted peripheral sympathoadrenal responses in HAAF to central nervous system mechanisms including: (1) the systemic-mediator hypothesis; (2) the brain fuel–transport hypothesis; (3) the brain-metabolism hypothesis; and (4) the cerebral-network hypothesis (rev. in (Cryer 2013b)). Still, the precise molecular mechanisms underlying the compromised autonomic defenses against recurrent hypoglycemia are not well understood.

In adult nondiabetic subjects (Davis et al. 2014), in patients with congenital hyperinsulinism (Christesen et al. 2012) and in normal rodents (Shum et al. 2001; Inouye et al. 2002; LaGamma et al. 2014), multiple-daily episodes of hypoglycemia can attenuate adrenal epinephrine secretion, which in turn, leads to hypoglycemia unawareness. However, the mechanisms underlying the reduced capacity of the adrenal glands to replenish its epinephrine stores in subjects with HAAF has not been studied in detail. We have shown that the blunted epinephrine responses in animals with counterregulatory failure correlate with decreased induction of adrenal TH mRNA (LaGamma et al. 2014), suggesting a possible down regulation of catecholamine biosynthesis.

It is well known that the transsynaptic release of catecholamines from the adrenal medulla in response to stress is accompanied by compensatory increases in catecholamine biosynthesis via mechanisms resulting in increased tyrosine hydroxylase (TH) protein and enzyme activity to maintain constant cellular catecholamine levels (Wakade et al. 1988). TH is the rate-limiting enzyme in catecholamine biosynthesis and its activity is upregulated by two main mechanisms: short-term (minutes) regulation is achieved through transient phosphorylation of existing TH protein molecules whereas long-term (several hours – days) regulation is achieved by increasing TH mRNA and protein (Dunkley et al. 2004). Therefore, changes in TH phosphorylation, TH mRNA, and total TH protein levels (which occur at differing time intervals following the onset of stress), are each influenced by adaptive neurohumoral physiological mechanisms known to be evoked during hypoglycemia (Dunkley et al. 2004; Lenartowski and Goc 2011). In this regard, acute glucose deprivation causes an increase in adrenal TH activity in the short term permitting replenishment of releasable catecholamine stores (Bobrovskaya et al. 2010). However, it is not known how or which of these systems are affected by exposure to recurrent hypoglycemia.

The approach taken here was to directly compare the sympathoadrenal responses (i.e., plasma epinephrine concentrations and adrenal capacity to produce catecholamines) to acute and recurrent hypoglycemia in normal, nondiabetic rats. Two experimental models of recurrent hypoglycemia were selected: (1) single daily episodes of hypoglycemia known to elicit normal stress responses (rev. in (Kvetnansky et al. 2009); and (2) twice daily antecedent episodes of hypoglycemia, shown by us and others to result in attenuated epinephrine responses (HAAF) (Shum et al. 2001; Inouye et al. 2002; LaGamma et al. 2014). All experimental groups (including the corresponding saline controls) were subjected to a final hyperinsulinemic-hypoglycemic clamp on day 4. The hypoglycemic clamp method was chosen because it provides a sustained and reproducible severity of hypoglycemia on the final day of the study prior to sacrifice. As an index of catecholamine biosynthesis, we quantified changes in the relative rate of TH transcription, steady-state TH mRNA levels, total TH protein and its site-specific phosphorylation at Ser40 – a marker of TH enzyme activation (Dunkley et al. 2004; Bobrovskaya et al. 2010; Lenartowski and Goc 2011). Counterregulatory responses were also monitored in order to assure proper implementation of the animal models.

## Materials and Methods

### Animals

Adult, male Sprague–Dawley rats with jugular vein (JV) and carotid artery (CA) catheter implants for stress-free infusion and blood collection were purchased from Harlan Labs, Inc. Indianapolis, IN. Animals were individually housed in temperature (22°C) and humidity-controlled rooms and allowed access to food (regular rat chow, Agway Prolab 3,000; Syracuse, NY) and water ad libitum unless otherwise specified. The animals were acclimated to the animal facility and handling for 3–4 days. Animal care and experimentation conformed to the Public Health Service Guide for Care and Use of Laboratory Animals and American Veterinary Medical Association Panel on Euthanasia Guidelines, and were approved by the Institutional Animal Care and Use Committee at New York Medical College and Yale University.

### Antecedent treatments

**Figure 1:**
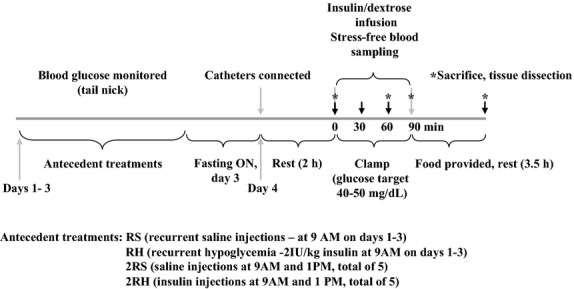
Schematic depicting the basic protocol used in these experiments.

The animals were randomly distributed into four experimental groups (each group consisted of at least six animals): sham-treated (recurrent saline injections) – either once daily (RS) or twice daily (at 9 am and 1 pm, 2RS) and subjected to recurrent insulin-induced hypoglycemia – either once daily (RH); or twice daily (2RH). Figure1 shows the timeline for the experiments. All treatments were for three consecutive days followed by a hyperinsulinemic-hypoglycemic glucose clamp on day 4. Antecedent hypoglycemic episodes were induced by intraperitoneal (i.p.) injection of regular human insulin (Humulin R, Eli Lilly, Indianapolis, IN) at a dose of 2 IU/kg body weight (Shum et al. 2001; LaGamma et al. 2014). Food was removed in all groups for 3 h after each insulin or saline injection. Blood glucose was monitored from tail nick samples using a handheld glucometer (AlphaTrak, Abbott Laboratories, Chicago, IL) every 30-min throughout each bout of hypoglycemia in RH and 2RH group in order to achieve and maintain glucose levels between 40 and 50 mg/dL. Hypoglycemia was terminated by providing the animals with solid food. In the 2RS and 2RH groups, only one injection was given on the morning of the third day. All animals were then fasted overnight before the glucose clamp studies on the 4^th^ day. Animal weight was monitored on a daily basis to ensure wellbeing and achievement of comparable nutrition.

### Hyperinsulinemic-hypoglycemic clamp

**Figure 2:**
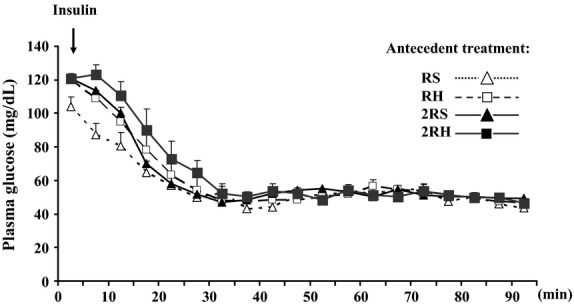
Plasma glucose concentrations during the hyperinsulinemic-hypoglycemic glucose clamp in once- and twice-daily saline control (RS and 2RS, respectively) and once- and twice-daily recurrent hypoglycemia groups (RH and 2RH, respectively). The values are shown as mean ± SE. *N* ≥ 6 for each experimental group.

On the day of the study, the vascular catheters were connected to extension sets for stress-free blood sampling (CA) and dextrose/insulin infusion (JV). Animals were rested at least 2 hrs before baseline sampling in order to let them recover from handling stress following connection to the infusion pumps. A constant infusion of regular human insulin (50 mIU/kg/min; Eli Lilly) and a variable 20% dextrose infusion were started at 0’ and plasma glucose levels were monitored every 5 min (GM9 Analyser, Analox Instruments Ltd, London, UK) to guide dextrose infusion. Plasma glucose levels were lowered to 45 mg/dL and maintained at this target until the end of the clamp study at 90’ (Fig.2) (LaGamma et al. 2014). Blood samples were collected at 30 min intervals throughout the study for subsequent measurement of plasma glucagon, catecholamine, and corticosterone concentrations. Following each sample collection, the erythrocytes were resuspended in an equivalent volume of artificial plasma and reinfused back into the animal through the carotid artery to prevent volume depletion and anemia (LaGamma et al. 2014). At the end of the clamp, blood glucose levels were recovered and the animals were killed 3.5 h later with an overdose of sodium pentobarbital given intravenously. The adrenal glands were rapidly removed, frozen, and stored in the −80°C freezer until analysis. We noted previously that the recovery of glucose levels following the clamp did not impact TH mRNA expression when compared with animals that were continuously maintained at hypoglycemic levels for 5 h (LaGamma et al. 2014). Subsets of animals were killed either before (0’) or during (30’ and 60’) the clamp procedure with an overdose of sodium pentobarbital, followed by decapitation. The adrenal medullae were dissected and immediately frozen on dry ice. The tissues were stored at −80°C until further analyses.

### Hormone analyses

Plasma hormone concentrations (glucagon, insulin, and corticosterone) were determined using commercially available radioimmunoassay kits from Linco Research, St. Charles, MO and Diagnostic Products, Los Angeles, CA (Chan et al. 2006). For measurement of catecholamine concentration, serum samples were analyzed using a competitive enzyme immunoassay (Rocky Mountain Diagnostics, Colorado Springs, CO) as described (Turcanu et al. 2012; LaGamma et al. 2014).

### Isolation of RNA and Northern blot analyses

Total RNA from each left AM was isolated using the RNeasy Mini kit (Qiagen, Valencia, ML) and RNA concentration and purity were evaluated by spectrometry at 260 and 280 nm using the NanoDrop 2000 (Thermo Fisher Scientific, Pittsburgh, PA). Changes in steady-state mRNA levels for the genes of interest were determined by northern blot as described previously (LaGamma et al. 2014). The blots were hybridized successively to labeled probes for TH, phenylethanolamine N-methyl transferase (PNMT), and 18S rRNA. Following exposure to X-ray film within the linear range of the signal, the autoradiographs were scanned and analyzed by Quantity One Software using BioRad GS 800 densitometer. The integrated optical density for each mRNA was normalized for the densities obtained for 18S rRNA levels in the same samples, on the same blot.

### RT-PCR analyses

The quantitative real-time RT-PCR assay was used to determine TH primary transcripts (an indirect measure of TH gene transcription rates) and steady-state TH mRNA levels from each adrenal total RNA sample following a RT reaction which was performed as described (Chen et al. 2008). The produced cDNAs (a 2 *μ*L aliquot from 20 *μ*L RT reaction) were subjected to real-time PCR amplification as recommended by the manufacturer (Applied Biosystems) using SYBR green indicator (Roche Diagnostics, Indianapolis, IN) and the following primer pair sets at 1 *μ*mol/L concentration: TH mRNA: forward – 620 5′- tcg gaa gct gat tgc aga ga-3′ and reverse – 695 5′- ttc cgc tgt gta ttc cac atg-3′and TH intron 2-specific: forward – 215 5′- gtt gct ctg gct agt gac ctg- 3′ and reverse – 284 5′-ggc cag gct gtc gat tct ggg a-3′. Glyceraldehyde 3-phosphate dehydrogenase (GAPDH) mRNA was measured in each adrenal sample for normalization purposes, using the same cDNA as that used for measuring TH mRNA or TH intron-2 primary transcripts (GAPDH primer set: forward – 507 5′- gga agg gct cat gac cac agt-3′ and reverse **-**570 5′- cca tca cgc cac agc ttt cca-3′)(Radcliffe et al. 2009). A routine melting curve analysis was performed after finishing the amplification to exclude the presence of nonspecific products. Data were calculated using the ΔΔCt method, normalized to GAPDH mRNA levels and expressed as relative fold change vs. control. GAPDH mRNA levels were not altered across the different experimental groups.

### Western blot analyses

Total protein extracts were prepared from each right adrenal medulla sample as described (Bobrovskaya et al. 2010). Protein lysates were separated on 10% SDS-PAGE, electroblotted onto a nitrocellulose membrane (BioRad; Hercules, CA) and incubated with primary antibody (P-Ser40 TH antibody at 1:1000, antibodies-online.com) overnight. After incubation with secondary antibody (Goat Anti-Rabbit IgG, from Pierce, Rockford IL; diluted 1:30 000) the immune reaction was visualized by enhanced chemiluminescent substrate from Pierce, utilizing a horseradish peroxidase label and Kodak XAR-5 film, as described by the manufacturer. Blots were reprobed with total TH antibody (at 1:4000 dilution; Imgenex, San Diego, CA) and primary antibody for housekeeping protein *β*-actin (1:2000, Monoclonal Anti-*β*-Actin antibody, Sigma, Saint Louis, MO) to confirm equal loading. Phospho-protein kinase A (PKA) substrate antibody from Cell Signaling Technology (Danvers, MA) was also used to determine activation of PKA signaling in the same lysates. The blots were exposed to autoradiography and the X-ray films were scanned and quantified with BioRad Quantity One software. For quantification, we always used a signal in the linear range. The ratios of TH/*β*-actin, PKA substrate/*β*-actin or P-TH/TH immunoreactivity were calculated for each sample and the results are presented as fold induction compared to the corresponding control group on the same Western blot.

### Statistics

Statistical analysis was performed with Sigma STAT/Plot software; version 12 (San Jose, CA). All data were expressed as means ± standard error of the mean (SEM). All differences were considered to be significant at *P* < 0.05. Comparisons of basal and hypoglycemic responses were made using the one way analysis of variance (ANOVA) followed by a Neuman–Keuls post hoc analysis. Sequential counterregulatory hormonal responses to hypoglycemia and glucose parameters during hypoglycemia were compared using repeated-measures ANOVA.

## Results

### Plasma glucose and insulin levels

During the glucose clamp, target plasma glucose levels (45 mg/dL) were achieved by 30 min and were maintained at that level in all groups (Fig.2). There were no statistically significant differences in the blood glucose levels between experimental groups at each time point during the hyperinsulinemic-hypoglycemic clamp. Also, no significant differences were observed in plasma insulin concentrations between the treatment groups at baseline or at the end of the glucose clamp study (Table1). These two observations demonstrate that all animals were exposed to identical glucose and insulin stimuli during the glucose clamp portion of the study, differing only by their antecedent history in the preceding 3 days.

**Table 1:** Plasma insulin concentrations on day 4 before (baseline) and after hyperinsulinemic-hypoglycemic clamp (hypo). Data are summarized from three independent experiments (*n* ≥ 6 per group); values are mean ± SE; units: uIU/mL

Group	RS	RH	2RS	2RH
Baseline	15 ± 4	11 ± 1	15 ± 5	16 ± 2
Hypo	4539 ± 305	4099 ± 690	4251 ± 221	3729 ± 245

### Twice daily recurrent hypoglycemia attenuated epinephrine and glucagon responses on day 4

**Figure 3:**
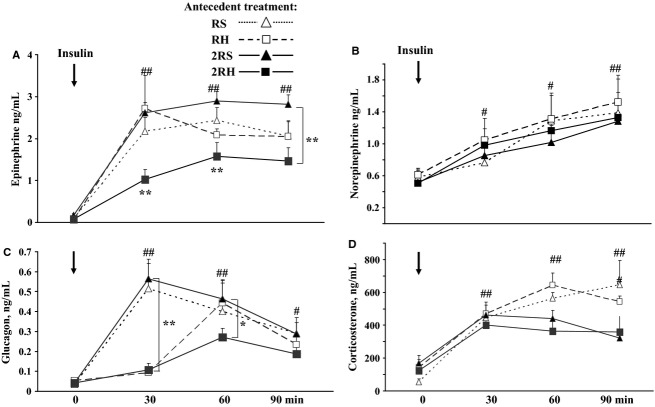
Plasma (A) epinephrine, (B) norepinephrine, (C) glucagon, and (D) corticosterone responses during the hypoglycemic clamp in once daily (RH) versus twice daily (2RH) recurrently hypoglycemic rats and the corresponding saline groups (RS and 2RS). Data are summarized from three independent experiments, *n* ≥ 6 animals per group. Values are shown as mean ± SE. ^#^*P* < 0.05 or ^##^*P* < 0.002 vs. 0 time point for each experimental group; *P < 0.05 or **P < 0.002 vs. corresponding control at given time point.

There were no statistically significant differences in baseline levels of epinephrine or glucagon across all treatment groups with regard to antecedent exposures to either recurrent saline or insulin injection (Fig.3). As expected, epinephrine and glucagon levels rose significantly from baseline values in all groups during the hypoglycemic clamp (##*P* < 0.002). Animals exposed to recurrent hypoglycemia (RH and 2RH) had delayed glucagon responses. In addition, the rise in both epinephrine (Fig.3A) and glucagon (Fig.3C) were attenuated by nearly 50% in the twice daily hypoglycemic stress group (2RH) compared to 2RS group (*P < 0.05). For epinephrine, both the peak values and area under the curve obtained for vehicle treated groups (RS and 2RS) and the once-daily hypoglycemic stress group (RH) did not differ from each other (3A). Plasma norepinephrine levels at baseline and the increments during the hypoglycemic clamp were not significantly different between the experimental groups (Fig.3B) and were consistent with previously published results (Shum et al. 2001; Kvetnansky et al. 2009). Glucocorticoid responses were also evaluated during the hypoglycemic glucose clamp (Fig.3D). The baseline values in all groups were not different from one another and showed the expected normal diurnal levels for that time of day (∼100 ng/mL). During the hypoglycemic clamp, plasma corticosterone levels increased in all groups (^#^*P* < 0.05 vs. corresponding baseline). Interestingly, with the increased frequency of stressful episodes (from once daily to twice daily) the twice-daily saline (2RS) and twice daily recurrent hypoglycemia groups (2RH) had lower corticosterone levels at later time points (60 and 90 min) compared to once-daily treated animals (RS and RH, *P < 0.05).

### Reduced adrenal catecholamine biosynthetic capacity following twice daily recurrent hypoglycemia

#### Limited induction of TH mRNA

**Figure 4:**
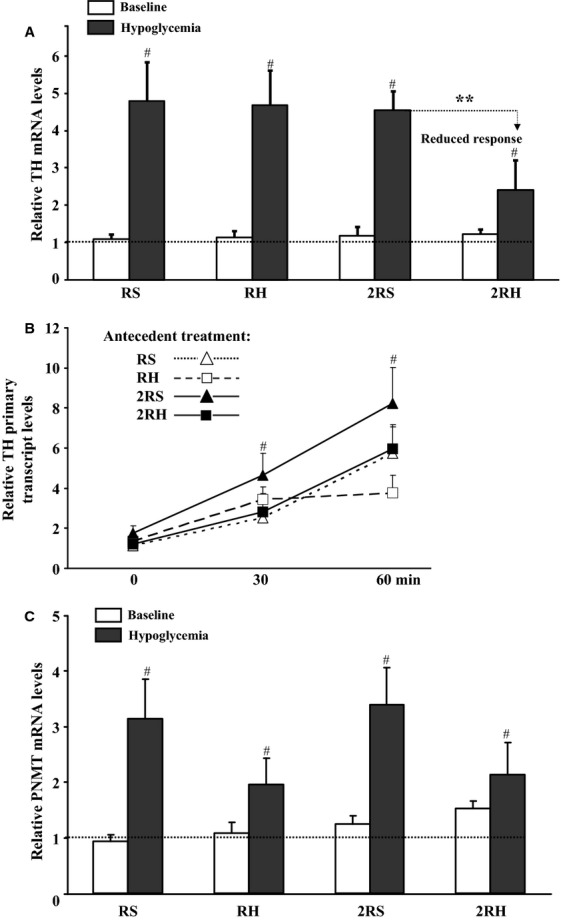
Effect of once daily (RH) versus twice daily (2RH) recurrent, antecedent hypoglycemic episodes on steady-state mRNA levels (A, C) and TH gene transcription (B) in nondiabetic rats. Total RNA was isolated from left adrenal medullae. The relative levels of TH (Fig.4A) and PNMT (Fig.4C) mRNA were determined by Northern blot analyses. Baseline – open bars (time matched controls, killed without hypoglycemic clamp on day 4; hypoglycemia (filled bars, animals underwent hypoglycemic clamp on day 4 and were killed 5 h after initiation of insulin infusion). Data are summarized from three independent experiments, *n* ≥ 6 animals per group. Values (mean ± SE) are shown as fold induction over unmanipulated animals, dotted line). ^#^*P* < 0.05 compared to unmanipulated animals; *P < 0.05 2RH compared to corresponding control (2RS). (B) The relative levels of TH primary transcripts in each experimental group were determined by real-time RT-PCR using TH intron 2-specific primers as described in methods. The obtained values for TH primary transcripts were divided by those for GAPDH mRNA measured in the same samples and expressed as fold-increase over baseline (0 time point). Data are summarized from two independent experiments in which set of animals were killed before (0 time point) and at 30 and 60 min after achieving target glucose levels during the hypoglycemic clamp, *n* = 6. ^#^*P *<* *0.05 compared to baseline.

As with prior work in this field (Vietor et al. 1996; Kvetnansky et al. 2009), and even after twice daily recurrent hypoglycemia (Fig.4, 2RH baseline), when adrenal levels of TH and PNMT mRNA were measured 24 h after the last dose of insulin or saline (without insulin-induced hypoglycemic clamp on day 4), they were not significantly different from unmanipulated control values (Fig.4A and C, open bars). After the hypoglycemic clamp, TH mRNA and PNMT levels increased in all groups (filled bars) relative to unmanipulated control values (dotted line) whether exposed to sham treatment (RS and 2RS experimental groups) or to antecedent hypoglycemia (RH and 2RH; ^#^*P* < 0.05; Fig.4A and C). However, the rise in TH mRNA was significantly less in the group exposed to twice daily episodes of antecedent hypoglycemia (2RH; *P < 0.05 vs. all other groups who underwent glucose clamp). This data suggest that defects in the adrenal medullary regenerative capacity for epinephrine lies upstream at the level of TH and not at the PNMT level. The reduction in TH mRNA in the 2RH group could be due to decreased activation of TH gene transcription or increased degradation of TH mRNA. Therefore, to determine whether transcriptional mechanisms contribute to the attenuated induction of TH mRNA following twice daily recurrent hypoglycemic episodes (2RH experimental group), we performed quantitative real-time RT-PCR analyses of total adrenal medullary RNA using TH intron 2-specific primers as described before (Chen et al. 2008). However, measuring the relative rate of TH transcription 5 h after initiation of insulin infusion on day 4 failed to reveal significant changes from baseline in all experimental groups (data not shown).

#### Increased relative rate of TH transcription

To establish whether the relative rate of TH gene transcription and TH activity were altered during acute and recurrent hypoglycemia, a modified protocol was used. In these experiments, sets of animals from each study group were killed before (0-time point) and during the hyperinsulinemic-hypoglycemic clamp on day 4 at 30 and 60 min after achieving the target plasma glucose levels. Counterregulatory hormone responses were monitored to ensure HAAF was induced after twice daily RH (data not shown). In all experimental groups, exposure to acute (RS and 2RS) or recurrent hypoglycemia (RH and 2RH) resulted in gradual increases of the relative rate of TH gene transcription (Fig.4B, ^#^*P* < 0.05). Notably, no statistically significant differences between the experimental groups were observed. At the examined time points (0, 30, and 60 min) adrenal steady-state TH mRNA levels remained unchanged in all groups as expected (data not shown).

#### Lack of accumulation of TH protein

**Figure 5:**
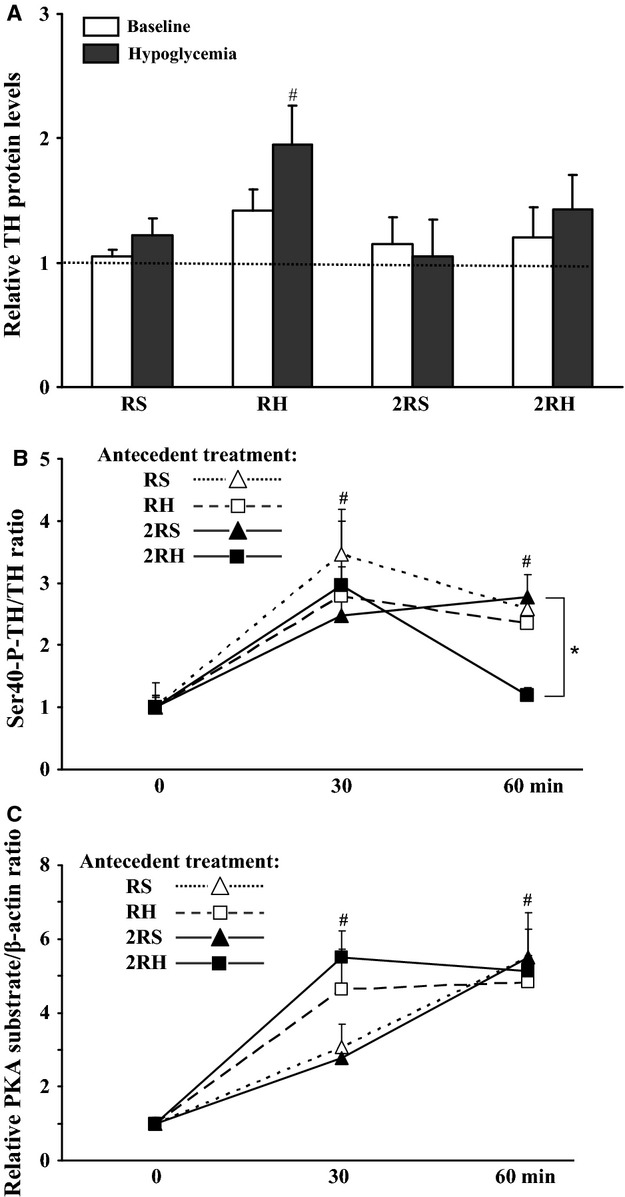
Effect of once daily (RH) versus twice daily (2RH) recurrent, antecedent hypoglycemic episodes on relative TH protein levels, P-Ser40 TH, and PKA activity in the adrenal medulla of normal, nondiabetic rats. Western blot analyses of total protein lysates from the right adrenal medulla of saline (animals subjected to single – RS or twice – 2RS daily injections of saline for 3 days, followed by glucose clamp on day 4); and RH groups (animals exposed to recurrent hypoglycemia once daily – RH or twice daily – 2RH for 3 days, followed by glucose clamp on day 4). (A) Baseline – open bars (time matched controls, animals were killed without hypoglycemic clamp on day 4); hypoglycemia – filled bars (animals were killed 5 h after infusion of insulin). Relative TH concentrations were calculated as a ratio of TH and *β*-actin immunoreactivity detected on the same blot. Data are summarized from three independent experiments, *n* ≥ 6 animals per group. Values (mean ± SE) are shown as fold induction over control (unmanipulated animals, dotted line). ^#^*P* < 0.05 compared to unmanipulated control group. (B) TH phosphorylation was calculated by dividing the amount of Ser40-phosphorylated TH by the amount of total TH for each lysate from individual adrenal medullae of animals killed during the hypoglycemic-hyperinsulinemic clamp (before and at 30 and 60 min after achieving target glucose levels). (C) Phospho-PKA substrate levels (quantified as optical density of all bands for each lane) were calculated relative to b-actin levels on the same blot. Data are summarized from *n* = 6 for each time point and presented as mean ± SE. ^#^*P* < 0.05 (vs. 0 time point), *P < 0.05 (2RH vs.2RS at 60 min).

The right adrenal medullae from the same animals were used for Western blot analyses as described in the methods section. In animals exposed to sham treatment (RS and 2RS experimental groups), total adrenal TH protein levels were not significantly different from baseline when examined 5 h after initiation of insulin infusion on day 4 (Fig.5A, open bars). These data are consistent with previous reports indicating that a longer time period or chronic exposure to stress is needed to alter the steady-state levels of TH protein (Vietor et al. 1996; Bobrovskaya et al. 2010). In contrast, a statistically significant increase in the relative levels of adrenal TH protein was found only in animals exposed to once-daily antecedent recurrent hypoglycemia (RH group, ^#^*P* < 0.05, Fig.5A), similar to the data reported before (Vietor et al. 1996). Importantly, TH protein following twice daily recurrent hypoglycemia regimen (2RH experimental group) was not significantly different from baseline or from its respective control (2RS, Fig.5A). Thus, the transient daily rise in TH mRNA levels was reflected in a statistically significant elevation of TH protein only following once-daily recurrent antecedent hypoglycemic episodes (RH group).

#### Activation of preexisting TH enzyme molecules for shorter period of time in 2RH group

The samples from the right AM were also subjected to western blot analyses using an antibody specific for TH protein phosphorylated at Ser40 (an index of TH enzyme activation, (Bobrovskaya et al. 2010; Dunkley et al. 2004). No differences between the experimental groups were observed at the 0 time point. Also when examined 5 hrs after the initiation of insulin infusion, the phospho-Ser40 TH protein levels were not different from baseline in all experimental groups (data not shown). However, the relative phospho-TH immunoreactivity increased significantly to a similar extent (2–3 fold over baseline) 30 min after achieving the target glucose levels in all groups (Fig.5B). It also remained significantly elevated at 60 min following exposure to acute (RS and 2RS groups) or once-daily recurrent hypoglycemia (RH group). Notably, in the 2RH group the relative Ser40 phospho-TH levels at 60 min were significantly lower (*P < 0.05 compared to 2RS group) and not different from baseline, suggesting that following the twice-daily recurrent hypoglycemia regimen, the duration of the adrenal TH enzyme activation in response to subsequent glucoprivic episodes is substantially reduced. There was no effect of acute or recurrent hypoglycemia or time (for up to 60 min) on total TH protein levels (data not shown).

A number of protein kinases have been found to phosphorylate TH at Ser40 in vitro and in situ, including PKA (Dunkley et al. 2004). To examine if insulin-induced hypoglycemia causes activation of adrenal PKA signaling, we performed western blot analysis on the same samples utilizing a phospho-PKA substrate specific antibody (Fig.5C). Phospho-PKA substrates appeared as multiple bands and increased significantly in number and intensity with time (0 to 60 min) in all experimental groups. These results suggest that adrenal PKA activation by acute and recurrent hypoglycemia may contribute to the observed increased Ser40 phospho-TH immunoreactivity.

## Discussion

Although previous studies have demonstrated a deleterious effect of antecedent hypoglycemia to reduce adrenal catecholamine secretion in response to a subsequent bout of hypoglycemia, the range of mechanisms underlying this defect have not been completely identified. This study is the first to assess both the short-term and long-term regulation of adrenal TH enzyme activity following prior exposure to insulin-induced hypoglycemia in normal rats. The major novel findings here are that in the 2RH group: (1) the duration of adrenal medullary TH enzyme activation is greatly reduced; (2) there is an elevated turnover of TH mRNA; and (3) there is a lack of accumulation of TH protein. All these factors indicate that the adrenal capacity to produce catecholamines is reduced in HAAF.

We aimed to identify the underlying mechanisms of reduced sympathoadrenal responses in HAAF by comparing two models of recurrent hypoglycemia: single daily-repeated stress model, (Vietor et al. 1996; Kvetnansky et al. 2009) and twice daily episodes of recurrent hypoglycemia (HAAF model, (Shum et al. 2001; Inouye et al. 2002; LaGamma et al. 2014) in normal nondiabetic rats. Consistent with prior work (DeCristofaro and LaGamma 1994; Vietor et al. 1996; Rusnak et al. 1998), we confirmed that once-daily recurrent hypoglycemic stress increased TH mRNA levels and TH protein content (Figs.4 and 5). TH activity (Ser40 TH phosphorylation, Fig.5B) was also elevated in the short-term, permitting resynthesis of epinephrine. These effects correlated temporally with a maximal counterregulatory hormonal response (Fig.3). In contrast twice daily episodes of recurrent antecedent hypoglycemia attenuated both TH mRNA ((LaGamma et al. 2014), Fig.4) and TH protein responses by ∼50% (Fig.5) similar to the reduced plasma epinephrine concentrations during HAAF (Fig.3). In addition, we demonstrate for the first time that the short-term regulation of TH activity was also affected in HAAF (see Fig.5B, 60 min).

Although semiquantitative in situ hybridization analysis of adrenals obtained from diabetic rats exposed to the same twice daily recurrent hypoglycemia paradigm (HAAF) demonstrated a reduction in TH and PNMT mRNA expression (Inouye et al. 2005, 2006), our more quantitative approach illustrated for the first time that repeated daily bouts of hypoglycemia can influence the molecular mechanisms that regulate adrenal catecholamine synthesis and limit its capacity to replenish the adrenal pool of epinephrine even in nondiabetic animals. Thus, the adrenal component of the HAAF phenomenon may not only be a direct result of the diabetic condition itself, but rather a consequence of a maladaptive chromaffin cellular stress response (Davis et al. 2014).

### TH gene expression-related mechanisms

The attenuated adrenal TH mRNA response following 2RH could be due to reduced induction of TH gene transcription, increased degradation of TH mRNA, or both. No significant differences in the relative TH gene transcription rate were observed between the experimental groups at baseline compared to the relative rate of transcription 3.5 hrs after termination of the glucose clamp. While similar progressive increases in the relative TH gene transcription rates were observed throughout the hypoglycemic clamp at 30 and 60 min under all conditions (Fig.4B), we did observe significantly reduced TH mRNA (Fig.4A) and protein levels (Fig.5A) in the 2RH group 5 h after the start of the insulin infusion. The failure to achieve maximal induction of biosynthesis (as evidenced by the attenuated adrenal TH mRNA and protein levels, Figs.4 and 5) appears to be a consequence of posttranscriptional control mechanisms that reduce TH mRNA longevity and translational potential (Wong and Tank 2007; Xu et al. 2007, 2009; Lenartowski and Goc 2011). Additional research will be necessary to ascertain whether any novel factors exist in vivo (e.g., lipid metabolites, hormones, etc.) that may evoke targeted degradation of TH mRNA in HAAF (Aranyi et al. 2007; Parab et al. 2007).

In related work, it has been reported that the epinephrine secretory response can be attenuated even after a single bout of hypoglycemia using higher doses of insulin (10 IU/kg, see (Flanagan et al. 2003)) than that used in this study or in Vietor et al. (1996) (5 IU/kg). These treatments can deplete epinephrine content by as much as 70% (Vollmer et al. 1992), indicating that not only the frequency of hypoglycemia exposure, but also the dose of insulin may be critical factors in the pathophysiology of HAAF. Whether the blunted plasma epinephrine response in the other HAAF models is also associated with a reduced capacity of the adrenals to produce catecholamines has not been established.

### Transsynaptic-dependent mechanisms

Given the fact that under HAAF conditions, adrenal sympathetic nerve impulse activity remains elevated for 24 h (Sivitz et al. 2001; Herlein et al. 2006), the attenuated plasma epinephrine responses and the reduced content of adrenomedullary catecholamines (Wilke and Hillard 1994; Herlein et al. 2006) must arise a priori from factors acting at the level of the chromaffin cell's capacity to sustain the releasable pool of catecholamines rather than from a failure of sustained sympathetic input.

During high-frequency adrenal nerve stimulation and after intense/severe or prolonged stress such as during insulin-induced hypoglycemia, epinephrine release and de novo synthesis are elevated due to the synergistic actions of both cholinergic-nicotinic and noncholinergic receptor activation by acetylcholine and colocalized presynaptic neuropeptides (Wakade 1988; Wakade et al. 1988; Haycock and Wakade 1992; Hamelink et al. 2002). In the high-dose insulin HAAF model, tachyphylaxis was suggested to be the etiologic mechanism of the failure to release epinephrine and to replenish its stores (Sivitz et al. 2001; Herlein et al. 2006). Our study does not support tachyphylaxis of cholinergic and noncholinergic adrenal receptors as in the 2RH (HAAF) group: (1) TH gene transcription rate remained elevated (Fig.4); (2) there was an initial rise in Ser40P-TH (Fig.5B, 30 min); and (3) phospho-PKA substrates (Fig.5C) were induced to a similar level in all experimental groups. Furthermore, tachyphylaxis was not observed using in situ models –that is, perfused rat adrenal glands had the capacity to sustain biosynthesis (increase TH mRNA, TH protein, and TH activity) and release of epinephrine for prolonged periods of time via transsynaptic-related mechanisms (Wakade 1988; Wakade et al. 1988). This suggests that there may be other transsynaptic or humoral factors (perhaps hormones or free fatty acids) that are activated during HAAF in vivo which might be critical for these changes in catecholamine biosynthesis (Hamelink et al. 2002; Aranyi et al. 2007; Parab et al. 2007; Wong and Tank 2007).

### Humoral-dependent mechanisms

In view of this, we also characterized the counterregulatory response to hypoglycemia by measuring plasma levels of corticosterone (Fig.3D). Although the plasma corticosterone responses demonstrated a progressive drop with increased frequency of stressful episodes (from once to twice daily), all animals exhibited significant elevations in corticosterone levels during hypoglycemia (Fig.3C) suggesting that glucocorticoids likely do not constitute a major reason for the attenuated adrenal TH mRNA response. Albeit, several studies in nondiabetic humans (rev. in (Davis et al. 2014)) and normal rats (Sandoval et al. 2003) showed that elevated plasma glucocorticoids during antecedent hypoglycemia may contribute to the development of HAAF centrally (Goldberg et al. 2006) this mechanism may not be affecting the adrenal medulla.

In contrast to TH mRNA levels, the frequency of recurrent daily hypoglycemia episodes did not significantly affect the magnitude of PNMT mRNA elevation after glucose clamp (Fig.4C). This is in agreement with previous reports (rev. in (Kvetnansky et al. 2009) and is also consistent with an intact glucocorticoid signal-transduction apparatus to the nucleus under all of our experimental conditions since only the TH mRNA elevation was significantly attenuated.

We also observed attenuated plasma glucagon levels during HAAF as previously reported (Beall et al. 2012; Cryer 2012). Glucagon arises from pancreatic *α*-cells which are regulated in a paracrine fashion by insulin and somatostatin secreted from neighboring *β*-cell and delta-cells, respectively, as well as by the firing of both sympathetic and parasympathetic nerve impulses (Ahren 2000; Ahren et al. 2006; Parekh 2009). Whether overlapping trans-synaptic and humoral biosynthetic mechanisms are shared by both glucagon and epinephrine have yet to be specified.

### TH-protein-dependent mechanisms

An intriguing question remains as to how TH protein and Ser40-TH phosphorylation are both decreased during HAAF? It has been shown previously, that a single episode of glucoprivation (i.p. injection of 2-deoxy-d-glucose) caused significant activation of the PKA and CDK signaling pathways in the adrenal medulla (followed by MAPK at later time points). This activation was associated with increased phosphorylation at Ser40-site of preexisting TH molecules in the short-term (Bobrovskaya et al. 2010), known to cause substantial increases in TH enzyme activity in vitro and in vivo (Dunkley et al. 2004; Ong et al. 2014). The results of this study newly document that similar events initially occur following both acute (RS and 2RS groups) and recurrent (RH and 2RH groups) insulin-induced hypoglycemia, Fig.5B and C. The changes in TH phosphorylation status were transient and not detectable by 5 hrs after initiation of insulin infusion. However, following twice daily recurrent hypoglycemia, the activation of TH enzyme was significantly reduced by 60 min into the clamp compared to all of the other treatment groups (Fig.5B), although other PKA-phosphorylated substrates remained elevated (Fig.5C). This suggests that the TH enzyme in the 2RH group can be activated to a similar extent as the other three treatment groups, but it is inactivated much more rapidly. Currently, there is no data regarding which protein phosphatase regulates TH activity in vivo by dephosphorylating Ser40 site. The only protein phosphatases known to dephosphorylate TH at Ser40 in vitro are PP2A and PP2C (Dunkley et al. 2004). However, some reports indicate that the phosphorylation of hydroxylases (including TH) is a prerequisite for proteasome-driven protein degradation in vitro (Iida et al. 2002; Kawahata et al. 2009; Nakashima et al. 2011). The potential contribution of adrenal ubiquitin proteasome system and endoplasmic reticulum stress (Woehlbier and Hetz 2011) in blunting the sympathoadrenal responses in HAAF has not been tested.

#### Limitations of design

The fact that we did not have sufficient tissue to simultaneously quantify adrenal content of catecholamines (one adrenal was used for RNA extraction and the other one was used for protein assays) is a limitation of our work. Nonetheless, reduced catecholamine content is a well-documented observation in HAAF (Wilke and Hillard 1994; Sivitz et al. 2001; Herlein et al. 2006). In future work, it will be useful to concurrently measure TH and PNMT enzymatic activity using the models we compared in this report.

#### Summary

We showed that antecedent twice-daily hypoglycemic events can attenuate the normal elevation in TH mRNA and TH protein levels, without affecting the relative rate of TH transcription in the adrenal medulla during a hypoglycemic clamp. In addition, the duration of TH enzyme activation is also shortened in HAAF. Taken together, our results suggest that in the setting of reduced insulin administration, the frequency of hypoglycemia (once vs. twice daily) alters the capacity of the adrenal medulla to replenish its releasable pool of catecholamines and may thus represent a significant adrenal medullary contribution to the neurogenic component of the clinical syndrome of HAAF. A better understanding of these underlying mechanisms may reveal novel therapeutic targets to treat HAAF.
